# Supplementary material for: Single and Repeated Oral Dose Toxicity and Genotoxicity of the Leaves of Butterbur
Source: Foods. 2021 Aug 23;10(8):1963. doi: 10.3390/foods10081963 (PMC8394925; doi:10.3390/foods10081963)
Supplement: Supplementary file 1 [file foods-10-01963-s001.zip › foods-1317906-supplementary.pdf]

Table S1. Summary of Body Weight and Food Intake of Sprague-Dawley Rats

Week	Group / Dose (mg/kg/day)			
	G1 / 0	G2 / 1,250	G3 / 2,500	G4 / 5,000
**Body weight (g)**				
**Sex : Male**				
0	211.7 ± 6.7	210.7 ± 6.8	211.7 ± 6.2	210.5 ± 3.5
4	364.7 ± 31.5	363.5 ± 26.9	373.6 ± 31.5	360.2 ± 21.0
8	520.5 ± 58.0	513.3 ± 214	514.7 ± 54.9	507.1 ± 40.5
13	605.1 ± 62.9	595.7 ± 67.1	624.3 ± 61.6	589.7 ± 47.1
**Sex: Female**				
0	159.6 ± 5.4	160.0 ± 5.8	159.8 ± 6.5	159.6 ± 8.5
4	227.1 ± 23.0	220.3 ± 9.0	222.8 ± 5.0	220.0 ± 23.8
8	295.3 ± 34.2	281.1 ± 12.4	282.9 ± 25.9	284.5 ± 32.6
13	336.2 ± 33.8	314.4 ± 19.9	312.9 ± 27.1	321.0 ± 39.3
**Food intake (g)**				
**Sex : Male**				
0	30.1 ± 2.1	30.0 ± 2.3	30.5 ± 3.3	29.6 ± 2.0
4	35.2 ± 3.6	36.2 ± 2.0	36.6 ± 4.8	34.8 ± 3.0
8	36.7 ± 3.7	37.1 ± 4.1	37.5 ± 4.0	35.8 ± 3.5
13	34.5 ± 3.4	34.6 ± 3.6	34.7 ± 3.0	33.5 ± 3.2
**Sex: Female**				
0	22.3 ± 1.9	23.1 ± 2.5	22.1 ± 2.3	23.1 ± 3.1
4	25.2 ± 3.1	23.9 ± 1.6	24.1 ± 1.7	24.7 ± 3.2
8	25.7 ± 2.3	24.2 ± 1.7	24.3 ± 2.0	25.3 ± 3.2
13	24.2 ± 1.3	22.5 ± 2.2	22.6 ± 2.5	23.9 ± 3.2

Table S2. Summary of Ophthalmological Examinations

Eye (Bilateral)	No. of animals	Findings	Group / Dose (mg/kg/day)			
			G1 / 0	G2 / 1,250	G3 / 2,500	G4 / 5,000
**Sex : Male**						
Pupil light reflex	5	Normal	5	5	5	5
Anterior segment	5	Normal	5	5	5	5
Transparent media	5	Normal	5	5	5	5
Fundus	5	Normal	5	5	5	5
**Sex: Female**						
Pupil light reflex	5	Normal	5	5	5	5
Anterior segment	5	Normal	5	5	5	5
Transparent media	5	Normal	5	5	5	5
Fundus	5	Normal	5	5	5	5

Table S3. Summary of Urinalysis Results

Urinalysis parameters		Group / Dose (mg/kg/day)			
		G1 / 0	G2 / 1,250	G3 / 2,500	G4 / 5,000
No. of animals		5	5	5	5
**Sex : Male**					
Volume (mL)		12.5 ± 3	14.0 ± 7	11.8 ± 6	15.7 ± 6
Color	Pale yellow	3	-	-	-
	Yellow	2	5	5	5
	Amber	-	-	-	-
Transparency	Clear	5	5	5	5
pH	7	2	-	-	-
	8	3	5	5	4
	9	-	-	-	1
Protein (mg/dL)	-	2	2	4	3
	25	2	2	1	2
	75	1	1	-	-
	150	-	-	-	-
	500	-	-	-	-
Glucose (mg/dL)	Normal	5	5	5	5
Ketone body (mg/dL)	-	2	-	-	-
	5	3	4	4	4
	15		1	1	1
Bilirubin (mg/dL)	-	5	5	5	5
Occult blood (Ery/μL)	-	3	5	5	5
	10	2	-	-	-
Cast	0	5	5	5	5
Epithelial cell	0	5	5	5	5
Leukocyte	0	5	5	5	5
Erythrocyte	0	2	5	5	5
	1~10	3	-	-	-
Specific gravity	1,021~1,030	-		1	-
	1,031~1,040	-	2	1	
	1,041~1,050	3	2	1	3
	1,051~1,060	1	-	3	2
	>1,060	1	1	-	-
**Sex : Female**					
Volume (mL)		5.4 ± 1.6	7.2 ± 2.8	6.6 ± 5.8	9.0 ± 5.2
Color	Pale yellow				
	Yellow	5	5	4	3
	Amber	-	-	1	2
Transparency	Clear	5	5	5	5
pH	7	-	-	-	-
	8	5	4	3	5
	9	-	1	2	-
Protein (mg/dL)	-	2	3	3	5
	25	3	2	2	-
	75	-	-	-	-
	150	-	-	-	-
	500	-	-	-	-
Glucose (mg/dL)	Normal	5	5	5	5
Ketone body (mg/dL)	-	1	-	-	-
	5	4	4	1	3
	15	-	1	3	2
	50	-	-	1	-
Bilirubin (mg/dL)	-	5	5	5	5
Occult blood (Ery/μL)	-	5	5	5	5
Cast	0	5	5	5	5
Epithelial cell	5	5	5	5	5
Leukocyte	0	5	5	5	5
Erythrocyte	0	5	5	5	5
	1~10	-	-	-	-
Specific gravity	1,021~1,030	-	-	1	2
	1,031~1,040	-	1	1	-
	1,041~1,050	1	1	-	1
	1,051~1,060	3	3	1	1
	>1,060	1	-	2	1

Table S4. Summary of Hematological and Clinical Chemistry Parameters

Parameter	Group / Dose (mg/kg/day)			
	**G1 / 0**	**G2 / 1,250**	**G3 / 2,500**	**G4 / 5,000**
**No. of animals**	**10**	**10**	**10**	**10**
**Sex : Male**				
RBC ($10^6/\mu L$)	8.68 ± 0.4	8.76 ± 0.3	8.49 ± 0.1	8.46 ± 0.3
HGB (g/dL)	15.9 ± 0.5	15.9 ± 0.4	15.5 ± 0.4	15.4 ± 0.3 *
HCT (%)	44.7 ± 1.3	44.8 ± 1.2	43.7 ± 0.9	43.4 ± 1.1 *
MCV (fL)	51.6 ± 1.9	51.1 ± 0.9	51.5 ± 1.4	51.3 ± 1.4
MCH (pg)	18.3 ± 0.8	18.2 ± 0.4	18.3 ± 0.6	18.2 ± 0.7
MCHC (g/dL)	35.5 ± 0.4	35.6 ± 0.2	35.5 ± 0.4	35.4 ± 0.6
PLT ($10^3/\mu L$)	1021 ± 81	980 ± 72	893 ± 132	936 ± 148
Reti (%)	2.87 ± 0.4	2.79 ± 0.3	3.09 ± 0.36	2.91 ± 0.2
WBC ($10^3/\mu L$)	9.78 ± 2.3	10.01 ± 2.1	9.32 ± 1.96	8.58 ± 2.0
NEU (%)	18.3 ± 5.5	20.0 ± 8.4	20.8 ± 6.9	21.3 ± 9.4
LYM (%)	70.8 ± 6.4	68.7 ± 8.7	67.6 ± 8.0	69.0 ± 9.7
MONO (%)	8.8 ± 2.0	9.7 ± 2.0	10.2 ± 2.1	8.3 ± 1.4
EOS (%)	1.8 ± 0.5	1.4 ± 0.4	1.3 ± 0.6 *	1.2 ± 0.4 *
BASO (%)	0.2 ± 0.1	0.2 ± 0.1	0.2 ± 0.1	0.2 ± 0.1
PT (sec)	19.3 ± 0.9	19.2 ± 0.5	19.1 ± 0.7	18.9 ± 0.9
APTT (sec)	15.4 ± 1.1	15.9 ± 1.9	15.8 ± 1.4	15.0 ± 2.3
ALT (U/L)	24 ± 3.5	30.7 ± 9.6	27.5 ± 4.1	26.7 ± 4.7
AST (U/L)	69.1 ± 8.4	76.1 ± 22.4	68.7 ± 8.5	73.3 ± 10.7
ALP (U/L)	245.3 ± 61.7	261.7 ± 43.6	235.2 ± 49.2	248.8 ± 70.5
GGT(U/L)	0.34 ± 0.1	0.37 ± 0.1	0.23 ± 0.1	0.31 ± 0.2
Glu (mg/dL)	134 ± 17	135 ± 13	132 ± 14	124 ± 12
BUN (mg/dL)	11.9 ± 1.2	12.1 ± 1.4	13.0 ± 2.0	12.5 ± 1.2
Crea (mg/dL)	0.45 ± 0.04	0.44 ± 0.05	0.46 ± 0.04	0.45 ± 0.03
T-bili (mg/dL)	0.08 ± 0.03	0.07 ± 0.03	0.06 ± 0.01	0.07 ± 0.03
T-Chol (mg/dL)	76 ± 15	93 ± 25	71 ± 16	80 ± 25
TG (mg/dL)	72 ± 20	82 ± 38	71 ± 31	74 ± 50
TP (g/dL)	5.8 ± 0.2	6.0 ± 0.2	5.9 ± 0.2	6.0 ± 0.3
Alb (g/dL)	2.3 ± 0.1	2.4 ± 0.1	2.3 ± 0.1	2.4 ± 0.1
A/G ratio	0.66 ± 0.1	0.66 ± 0.04	0.64 ± 0.04	0.65 ± 0.04
P (mg/dL)	5.62 ± 0.4	5.44 ± 0.6	5.69 ± 0.3	6.15 ± 0.44 *
Ca (mg/dL)	9.9 ± 0.4	10.2 ± 0.4	10.2 ± 0.3	10.1 ± 0.3
Na (mmol/L)	139.1 ± 1.5	138.6 ± 0.7	138.5 ± 1.3	137.5 ± 2
K (mmol/L)	3.89 ± 0.2	3.95 ± 0.2	3.78 ± 0.2	3.87 ± 0.15
CI (mmol/L)	107.8 ± 1.7	106.8 ± 0.8	106.9 ± 1.6	105.5 ± 2.1 **
**Sex: Female**				
RBC ($10^6/\mu L$)	8.19 ± 0.5	8.21 ± 0.4	7.71 ± 0.55 *	7.94 ± 0.3
HGB (g/dL)	15.6 ± 0.8	15.8 ± 0.4	15.4 ± 0.5	15.3 ± 0.6
HCT (%)	42.8 ± 2.1	43.6 ± 1.1	42.4 ± 1.2	42.4 ± 1.4
MCV (fL)	52.3 ± 1.2	53.2 ± 1.6	55.1 ± 3.0 #	53.4 ± 1.2
MCH (pg)	19.0 ± 0.5	19.3 ± 0.6	20.0 ± 0.9 **	19.3 ± 0.6
MCHC (g/dL)	36.3 ± 0.2	36.2 ± 0.2	36.3 ± 0.4	36.2 ± 0.6
PLT ($10^3/\mu L$)	901 ± 30	928 ± 62	903 ± 165	890 ± 99
Reti (%)	2.55 ± 0.3	2.37 ± 0.4	3.54 ± 2.4	2.28 ± 0.4
WBC ($10^3/\mu L$)	5.78 ± 3.3	5.15 ± 1.1	5.22 ± 1.2	4.70 ± 1.9
NEU (%)	19.8 ± 5.4	17.0 ± 6.9	14.5 ± 6.9	18.2 ± 4.6
LYM (%)	69.7 ± 7.0	73.3 ± 7.2	76.6 ± 7.7	71.4 ± 5.1
MONO (%)	8.4 ± 1.7	8.0 ± 1.8	7.4 ± 2.1	8.4 ± 0.8
EOS (%)	1.8 ± 0.6	1.4 ± 0.4	1.2 ± 0.6	1.7 ± 0.7
BASO (%)	0.3 ± 0.2	0.2 ± 0.1	0.3 ± 0.1	0.3 ± 0.1
PT (sec)	18.1 ± 0.8	18.6 ± 0.7	18.6 ± 0.9	19.0 ± 1.0
APTT (sec)	16.0 ± 0.8	15.0 ± 1.7	15.5 ± 0.9	15.4 ± 0.7
ALT (U/L)	26.2 ± 9.5	24.8 ± 7.0	20.7 ± 4.1	20.8 ± 4.8
AST (U/L)	64.9 ± 14.3	63.6 ± 11.6	65.1 ± 13.2	60.3 ± 11.9
ALP (U/L)	134 ± 35.5	128.7 ± 40.8	126.2 ± 47.2	113.2 ± 41.3
GGT(U/L)	0.53 ± 0.3	0.47 ± 0.2	0.52 ± 0.3	0.56 ± 0.24
Glu (mg/dL)	133 ± 12	126 ± 16	128 ± 22	122 ± 13
BUN (mg/dL)	11.7 ± 1.8	12.6 ± 1.9	11.9 ± 2.4	11.8 ± 2.1
Crea (mg/dL)	0.52 ± 0.04	0.53 ± 0.07	0.51 ± 0.05	0.47 ± 0.05
T-bili (mg/dL)	0.09 ± 0.03	0.10 ± 0.02	0.10 ± 0.03	0.07 ± 0.02

T-Chol (mg/dL)	102 ± 11	94 ± 13	85 ± 14 *	82 ± 19 *
TG (mg/dL)	26 ± 22	16 ± 8	18 ± 4	20 ± 8
TP (g/dL)	6.4 ± 0.5	6.1 ± 0.4	6.1 ± 0.3	6.1 ± 0.4
Alb (g/dL)	2.9 ± 0.3	2.7 ± 0.2	2.7 ± 0.3	2.7 ± 0.2
A/G ratio	0.82 ± 0.1	0.79 ± 0.05	0.79 ± 0.07	0.79 ± 0.06
P (mg/dL)	4.05 ± 0.9	4.80 ± 0.9	4.64 ± 0.7	4.73 ± 0.6
Ca (mg/dL)	9.9 ± 0.7	9.9 ± 0.3	9.9 ± 0.3	9.7 ± 0.3
Na (mmol/L)	138.7 ± 1.0	138.6 ± 0.8	139.1 ± 1.1	137.6 ± 1.0 *
K (mmol/L)	3.62 ± 0.2	3.71 ± 0.3	3.70 ± 0.3	3.81 ± 0.3
CI (mmol/L)	107.8 ± 1.4	107.7 ± 0.7	109.2 ± 1.0 *	107.7 ± 1.1

Significantly different from control by Dunnett's t-test : * p<0.05, **p<0.01
Significantly different from control by Steel test : # p<0.05

Table S5. Summary of Mean Absolute Organ Weights

Week	Group / Dose (mg/kg/day)			
	**G1 / 0**	**G2 / 1,250**	**G3 / 2,500**	**G4 / 5,000**
**Organ weight (g)**				
**Sex :Male**				
Body weight	590.8 ± 62.1	586.2 ± 70.3	611.8 ± 63.3	576.0 ± 46.5
Brain	2.21 ± 0.05	2.17 ± 0.08	2.23 ± 0.05	2.17 ± 0.04
Pituitary gland	0.0135 ± 0.0016	0.0126 ± 0.0020	0.0123 ± 0.0015	0.0134 ± 0.0021
Heart	1.68 ± 0.17	1.65 ± 0.18	1.64 ± 0.12	1.60 ± 0.15
Lung	1.77 ± 0.16	1.73 ± 0.19	1.79 ± 0.12	1.72 ± 0.19
Liver	14.96 ± 1.48	16.03 ± 2.86	16.63 ± 2.80	16.71 ± 2.34
Spleen	0.92 ± 0.16	0.87 ± 0.12	0.99 ± 0.19	0.86 ± 0.11
Kidney	3.40 ± 0.22	3.54 ± 0.39	3.62 ± 0.27	3.74 ± 0.28
Adrenal gland	0.0679 ± 0.00857	0.0682 ± 0.0070	0.0618 ± 0.0084	0.0632 ± 0.0070
Testis	3.45 ± 0.73	3.68 ± 0.36	3.56 ± 0.61	3.66 ± 0.37
Prostate	0.71 ± 0.12	0.73 ± 0.17	0.63 ± 0.16	0.67 ± 0.12
**Organ weight (g)**				
**Sex: Female**				
Body weight	326.0 ± 33.3	304.8 ± 21.0	299.3 ± 29.2	309.3 ± 40.0
Brain	1.97 ± 0.06	1.98 ± 0.09	1.98 ± 0.10	2.02 ± 0.13
Pituitary gland	0.0187 ± 0.0055	0.0184 ± 0.0031	0.0174 ± 0.0039	0.0168 ± 0.0022
Heart	1.04 ± 0.08	0.97 ± 0.08	0.97 ± 0.07	0.99 ± 0.10
Lung	1.27 ± 0.08	1.26 ± 0.05	1.24 ± 0.13	1.29 ± 0.12
Liver	8.31 ± 0.75	7.48 ± 0.49	7.85 ± 0.76	7.97 ± 1.12
Spleen	0.60 ± 0.06	0.53 ± 0.05	0.57 ± 0.07	0.57 ± 0.08
Kidney	1.97 ± 0.18	1.89 ± 0.15	1.92 ± 0.15	2.11 ± 0.29
Adrenal gland	0.0706 ± 0.0154	0.0642 ± 0.0117	0.0728 ± 0.0085	0.0692 ± 0.0077
Ovary	0.0877 ± 0.0149	0.0812 ± 0.0229	0.0991 ± 0.0094	0.0909 ± 0.0185
Uterus	0.58 ± 0.21	0.66 ± 0.18	0.66 ± 0.26	0.55 ± 0.11

Table S6. Result of Main Study in Male ICR Mice

Group	Dose	Route	Hours after dosing	Animal ID	PCE / (PCE+NCE)	MNPCE / PCE
Negative control (Water for injection)	0	P.O.	24	1101	149 / 500	0 / 4,000
				1102	149 / 500	2 / 4,000
				1103	143 / 500	2 / 4,000
				1104	146 / 500	3 / 4,000
				1105	150 / 500	1 / 4,000
				Total	737 / 2,500	8 / 20,000
				%(Mean ± S.D.)	29.5 ± 0.58	0.040 ± 0.029
Test Substance (KP-1)	1,250	P.O.	24	1201	152 / 500	3 / 4,000
				1202	148 / 500	2 / 4,000
				1203	146 / 500	2 / 4,000
				1204	155 / 500	0 / 4,000
				1205	154 / 500	1 / 4,000
				Total	755 / 2,500	8 / 20,000
				%(Mean ± S.D.)	30.2 ± 0.77	0.040 ± 0.029
	2,500	P.O.	24	1301	145 / 500	0 / 4,000
				1302	157 / 500	2 / 4,000
				1303	158 / 500	2 / 4,000
				1304	152 / 500	3 / 4,000
				1305	161 / 500	2 / 4,000
				Total	773 / 2,500	9 / 20,000
				%(Mean ± S.D.)	30.9 ± 1.25	0.045 ± 0.027
	5,000	P.O.	24	1401	144 / 500	1 / 4,000
				1402	139 / 500	1 / 4,000
				1403	144 / 500	1 / 4,000
				1404	148 / 500	2 / 4,000
				1405	150 / 500	1 / 4,000
				Total	725 / 2,500	6 / 20,000
				%(Mean ± S.D.)	29.0 ± 0.85	0.030 ± 0.011
Positive Control (MMC)	2	I.P	24	1501	168 / 500	286 / 4,000
				1502	171 / 500	251 / 4,000
				1503	153 / 500	244 / 4,000
				1504	164 / 500	218 / 4,000
				1505	179 / 500	256 / 4,000
				Total	835** / 2,500	1,255## / 20,000
				%(Mean ± S.D.)	33.4 ± 1.91	6.275 ± 0.611

P.O.: Per Os., I.P.: Intraperitoneal, MNPCE: Micronucleated polychromatic erythrocyte,
PCE: Polychromatic erythrocyte, NCE: Normochromatic erythrocyte, MMC: Mitomycin C
Significantly different from control by Aspin-Welch t-test : ** $p<0.01$,
Significantly different from control by Mann-Whiney test : ## $p<0.01$

Table S7. Summary of Micronucleus Test

Historical control values of MNPCE

Group	Hours after dosing (hr)	Dose (mg/kg)	N	MNPCE / PCE (%) (Mean ± S.D.)	Range[1] [MNPCE / PCE] (%)		95 % control limit [MNPCE / PCE][2]
					MIN	MAX	
Negative control	24	0	32	0.042 ± 0.019	0.007	0.077	<13
Positive control	24	2	32	6.119 ± 1.275	4.988	7.250	-

Historical control values of ratio of PCE to total erythrocytes

Group	Hours after dosing (hr)	Dose (mg/kg)	N	MNPCE / PCE (%) (Mean ± S.D.)	Range [MNPCE / PCE] (%)	
					MIN	MAX
Negative control	24	0	32	30.66 ± 3.006	25.96	35.36
Positive control	24	2	32	29.39 ± 3.864	24.72	34.07

Negative control: Water for injection, Normal saline injection, Corn oil, 0.5 % methylcellulose 1500 centripoise solution, 0.5% carboxymethylcellulose sodium salt soluton, *etc.*

Positive control: Mitomycin C (2 mg/kg, I.P., single dosing)

[1]The range was calculated by the control limit of X derived from X-R value.

[2]Poisson-based 95 % control limits of the historical negative control data.

Table S8. Summary of Main Study

Test substance	Dose (μg/mL)	RPD (%)	S9 mix	Trt-Ree time (hr)	No. of cell analyzed	Number of cells with structural aberrations									Number of cells with numerical aberrations			Others[1]
						ctd	csb	cte	cse	frg	gap		Total (%)		end	pol	Total (%)	
											ctg	csg	gap-	gap+				
Water for injection	0	100	-	6-18	150	0	0	0	0	0	0	0	1 (0.3)	1 (0.3)	0	1	1 (0.3)	0
					150	0	0	1	0	0	0	0			0	0		
KP-1	78.1	98.1	-	6-18	not observed													
	156	96.2	-	6-18	150	0	0	0	0	0	0	0	0 (0.0)	0 (0.0)	0	0	0 (0.0)	0
					150	0	0	0	0	0	0	0			0	0		
	313	93.8	-	6-18	150	0	0	0	0	0	0	0	0 (0.0)	0 (0.0)	0	0	0 (0.0)	0
					150	0	0	0	0	0	0	0			0	0		
	625	90.3	-	6-18	150	0	0	0	0	0	1	0	0 (0.0)	1 (0.3)	0	0	0 (0.0)	0
					150	0	0	0	0	0	0	0			0	0		
MMC	0.1	58.7	-	6-18	150	13	0	42	0	0	0	0	86** (28.7)	86 (28.7)	0	0	0 (0.0)	0
					150	15	2	40	0	0	2	0			0	0		
Water for injection	0	100	+	6-18	150	0	0	1	0	0	1	0	1 (0.3)	2 (0.7)	0	0	1 (0.3)	0
					150	0	0	0	0	0	0	0			0	1		
KP-1	78.1	93.0	+	6-18	not observed													
	156	87.2	+	6-18	150	0	0	1	0	0	0	0	1 (0.3)	1 (0.3)	0	0	1 (0.3)	0
					150	0	0	0	0	0	0	0			0	1		
	313	86.1	+	6-18	150	0	0	0	0	0	0	0	0 (0.0)	0 (0.0)	0	0	0 (0.0)	0
					150	0	0	0	0	0	0	0			0	0		
	625	82.1	+	6-18	150	0	0	0	0	0	0	0	0 (0.0)	0 (0.0)	0	0	1 (0.3)	0
					150	0	0	0	0	0	0	0			0	1		
B[*a*]P	20	51.4	+	6-18	150	10	0	25	1	0	0	0	60** (20.0)	60 (20.0)	0	0	0 (0.0)	0
					150	7	0	28	0	0	2	0			0	0		
Water for injection	0	100	-	24-0	150	1	0	0	0	0	0	0	1 (0.3)	1 (0.3)	0	1	1 (0.3)	0
					150	0	0	0	0	0	0	0			0	0		
KP-1	78.1	92.1	-	24-0	not observed													
	156	89.7	-	24-0	150	0	0	0	0	0	0	0	0 (0.0)	0 (0.0)	0	0	0 (0.0)	0
					150	0	0	0	0	0	0	0			0	0		
	313	85.1	-	24-0	150	0	0	0	0	0	0	0	1 (0.3)	1 (0.3)	0	1	1 (0.3)	0
					150	1	0	0	0	0	0	0			0	0		
	625	79.6	-	24-0	150	0	0	0	0	0	0	0	0 (0.0)	0 (0.0)	0	1	1 (0.3)	0
					150	0	0	0	0	0	0	0			0	0		
MMC	0.1	52.6	-	24-0	150	25	0	60	0	0	0	0	125** (41.7)	125 (41.7)	0	0	0 (0.0)	0
					150	21	1	52	0	0	6	0			0	0		

Aberration: ctg: chromatid gap, csg: chromosome gap, ctb: chromatid break, cte: chromatid exchange, csb: chromosome break, cse: chromosome exchange, frg: fragmentation, end: endoreduplication, pol: polyploidy

MMC: Mitomycin C, B[*a*]P: Benzo[*a*]pyrene, RPD: Relative Population Doubling, Trt-Rec time: Treatment-Recovery times, gap-:Total number of cells with structural aberrations excluding gap, gap+: Total number of cells with structural aberrations including gap

[1]Other were excluded from the number of cells with chromosomal aberrations.

Significant difference from negative control by Fisher's extract test: ** $p<0.01$ #: precipitation

Table S9. Historical Control Data

Historical control values of structural aberrations								
Group	S9 mix	Trt-Rec time (hr)	N	Structural aberration cells excluding gap (%) (Mean ± S.D.)	Range (%)		95 % control limit[3] [Structural aberration cells/300 cells]	
					MIN	MAX	MIN	MAX
Negative	-	6-18	44	0.288 ± 0.364	0	1.01*	0	<3
	+	6-18	44	0.311 ± 0.390	0	1.09*	0	<3
	-	24-0	42	0.246 ± 0.361	0	0.87*	0	<2
Positive	-	6-18[1]	39	23.44 ± 5.667	11.09*	35.78*		
	+	6-18[2]	39	24.64 ± 4.922	12.13*	37.15*		
	-	24-0[1]	37	35.37 ± 6.862	19.09*	51.65*		

Historical control values of ratio of PCE to total erythrocytes								
Group	S9 mix	Trt-Rec time (hr)	N	Numerical aberration cells (%) (Mean ± S.D.)	Range (%)		95 % control limit[3] [Structural aberration cells/300 cells]	
					MIN	MAX	MIN	MAX
Negative	-	6-18	44	0.174 ± 0.292	0	0.83*	0	<2
	+	6-18	44	0.167 ± 0.264	0	0.97*	0	<2
	-	24-0	42	0.262 ± 0.290	0	1.13*	0	<2

Negative control: Water for injection, Dimethyl sulfoxide, Acetone,

Trt-Rex time: Treatment-Recovery times

[1]Mitomycin C (0.1 μg/mL), [2]Benzo[*a*]pyrne (20 μg/mL), [3]Poisson-based 95 % control limits of the historical negative control data.

N: The total number of chromosome aberration test

The above historical control values were obtained from the data pooled from Jul. 15, 2013 to May 22, 2017.

*The range was calculated by the control limit of X derived from X-R-Rs value.
